# A political ontology for Europe: Roberto Esposito’s instituent paradigm

**DOI:** 10.1007/s11007-021-09542-z

**Published:** 2021-03-27

**Authors:** Rita Fulco

**Affiliations:** Classe di Lettere e Filosofia, Scuola Normale Superiore, Pisa, Italy

**Keywords:** Europe, Instituent thought, Political ontology, Negation, Conflict, Roberto Esposito

## Abstract

The aim of my article is to relate Roberto Esposito’s reflections on Europe to his more recent proposal of *instituent thought*. I will try to do so by focusing on three theoretical cornerstones of Esposito’s thought: the first concerns the evidence of a link between Europe, philosophy and politics. The second is deconstructive: it highlights the inadequacy of the answers of the most important contemporary ontological-political paradigms to the European crisis, as well as the impossibility of interpreting this crisis through theoretical-political categories such as *sovereignty*. The third relates more directly to the proposal of a new political ontology, which Esposito defines as *instituent thought*. Esposito’s discussion of political theology is the central theoretical nucleus of this study. This discussion will focus, in particular, on the category of *negation*, from which any political ontology that is based on *pure affirmativeness* or *absolute negation* is criticized. In his opinion, philosophical theories developed on the basis of these assumptions have proved to be incomplete or ineffective in relation to the current European and global philosophical and political crisis. Esposito therefore perceives the urgent need to propose a line of thought that is neither negatively destituent (post-Heideggerian), nor affirmatively constituent (post-Deleuzian, post-Spinozian), but instituent (neo-Machiavellian), capable of thinking about *order* through *conflict* (the affirmative through the negative). Provided that we do not think of the institution *statically*–in a conservative sense–but *dynamically*, as constant *instituting* in which conflict can become an instrument of a politics increasingly inspired by justice.

## A premise: dealing with political theology

The goal of my article is to relate Roberto Esposito’s reflections on Europe to his more recent proposal of *instituent thought*.

I will try to do so by focusing on three theoretical cornerstones of Esposito’s thought: the first concerns the evidence of a link between Europe, philosophy and politics. The second is deconstructive: it highlights the inadequacy of the answers of some the most important contemporary ontological-political paradigms to the European crisis, as well as the impossibility of interpreting this crisis through theoretical-political categories such as *sovereignty*. The third relates more directly to the proposal of a new political ontology, which Esposito defines as *instituent thought* within a neo-Machiavellian paradigm.

Before analytically addressing individual questions, I believe it is necessary to highlight certain points of what constitutes, in my opinion, a theoretical turning point that is important to understanding Roberto Esposito’s most recent thought, namely the path that leads him to the thesis of *Politics and Negation*.^1^ It is important to clarify, in this regard, that the volume that precedes it chronologically *A Philosophy for Europe: From the Outside* is not the one that precedes it theoretically.^2^ Its theoretical antecedent is in fact *Two: The Machine of Political Theology and the Place of Thought* in which Esposito deals directly with the question of *political theology*, which, as we know, has always been one of the main subjects of his criticism.^3^ In his opinion, despite the fact that political theology has been central to the complex and wide-ranging philosophical-political debate throughout the twentieth century, there are still many unresolved questions related to it.^4^ Indeed, political theology is the perspective within which, today, we find ourselves immersed and whose language we use, as is evident from the fact, already stressed by Schmitt, that all the great legal-political categories–sovereignty, ownership, freedom and people–are pervaded by it. Consequently, we cannot avoid the impression of being unable to assume, with regard to the theological-political perspective, the distance that would be necessary for effective criticism: “All the categories that have been employed on various occasions to arrive at the connection between politics and theology–like “disenchantment” or “secularization” or “profanation”–turn out to have political-theological origins themselves. By this I mean that they presuppose what they should explain.”^5^ However, all the categories proposed to overcome it–secularization, profanation–are also found, according to Esposito, inside it, partly because the interpreters that try to get out of it in fact remain imprisoned. Why does this happen? To take a prime example, which concerns the famous theological-political debate between Schmitt, Peterson and Taubes, Esposito believes that the attempt by each of them to overcome the categories of the other actually leads them to insert these categories within their own system of thought, without, therefore, really overcoming them.^6^ Esposito’s thesis is that “this process of exclusionary assimilation is the fundamental, defining action of the political-theological machine. It operates precisely by separating what it purports to join and by unifying what it divides, by submitting one part to the domination of the other.”^7^ In these categories, therefore, the *negative* definition prevails over the *positive* one: in Schmitt’s classic opposition between *friend* and *enemy*, for example, friend is simply characterized as *non-enemy*.^8^ In reality, the *positive* connotation of friend attributes to it something *much more* than being a simple non-enemy: precisely this positivity, in Schmitt’s thought, will always remain in the shade.^9^ This outcome emerges quite clearly in the different variations of one of the categories on which Esposito, starting from his previous research, has focused his attention, namely that of *person*, which, in his opinion, assumes the importance of a real theological-political *apparatus*.^10^ In the *person* category, in fact, there remains a *binary* opposition of parts, one of which is necessarily subjected to the other, whether the soul and the body or the rational part and the passionate part. This binary opposition, typical of Roman law and Christianity, from which the notion of person derives, is inherited, in his opinion, from the logic of “inclusive exclusion” that characterizes the theological-political machine. To counter this logic, starting in the last part of *Two*, Esposito tries to fathom different paths, such as that of Nietzsche, Spinoza, Bergson and Deleuze who, finding original ways of articulating subjectivity and thought, fractured, in his view, the person’s apparatus inherited from political theology.^11^ Deleuze, in particular, suggests a possible departure from the theological-political perspective. The fact that Deleuze does not explicitly deal with political theology is because he directly identifies it with the capitalist machine and indeed, according to Esposito, his main merit in this regard is precisely the “identification of the economic consequence of the political-theological dispositif, already implicit in the oikonomic matrix of the category of person.”^12^ Rather than countering the theological-political machine from the outside, Deleuze believes that it should be deconstructed from within: “Rather than attempt, in vain, to escape from the single dimension in which we are caught, Deleuze proposes reconverting it, by freeing the potential energy residing within it.”^13^ The proposal of a *radical immanence* and an *impersonal* subject, theorized in Deleuze’s final works, constitute, according to Esposito, an alternative to the logic of inclusive exclusion that fuels the theological-political apparatus. The conclusions of *Two* are therefore still rather close to those of *Bios*, in which the greatest inspiration for the thesis of a possible *affirmative* form of biopolitics was Deleuze’s last works and the authors to whom he refers.^14^

However, it was precisely with respect to radical Deleuzian immanence that, in *Politics and Negation*, a change of perspective and a departure from that affirmativeness without residue began, through a radical reflection on the question of *negation*. It was this genealogical research on the role of negation in the Western theological-political perspective that led Esposito to directly reflect on the question of political ontology and institutions, as an explicit philosophical and political response to the political-institutional crisis affecting not only Europe, but the whole world. Once again it is important to point out that the next step following *Politics and Negation* was not so much the second volume of *Terms of the Political* which immediately succeeded it chronologically, but the article that opens the first issue of the *Almanacco di Filosofia e Politica* of which Esposito is both the founder and director.^15^ The crisis to which Esposito intends to respond, including through this publishing project, is, first of all, that of the left, but also, more generally, that of the ways in which politics is conceived. This is why Esposito imagined, firstly, in the pages of the *Almanacco*, a philosophical *agora*: philosophy can and must play a special role in this challenge, since its task will be to rethink itself and, at the same time, to try to understand the deep meaning of the crisis; To seek to define it in its causes, including theoretical causes, and to address it. A philosophical and political project, therefore, that anticipates the fundamental themes of the volumes on political ontology.^16^

In the essay *Pensiero istituente. Tre paradigmi di ontologia politica* there appears to be a turning point in Esposito’s philosophical-political path. The key theme is in fact that of *instituent thought*. In it, as we shall see later, certain categories are taken into consideration that deal with the negative, without being crushed by it, but rather assuming it affirmatively. It is no coincidence that this further movement of thought took place at a particular *juncture*, namely in conjunction with the current historical phase during which at least three fundamental hypotheses have failed: firstly, that of an indefinite development of democracy, secondly, that of globalization without residue, and finally, that of the disappearance of borders.^17^ It was precisely these illusions, according to Esposito, which determined the left’s incapacity to grasp the severity of the crisis and consequent failure to equip themselves to deal with it. Philosophically, the most important proposals–which would have been a valid answer to it–namely, in particular, weak thought, hermeneutics and deconstruction, despite their valuable analysis, have proved, according to Esposito, incapable of really responding to the crisis.

Starting from this lacking, or ineffective, response, Esposito perceives the urgent need to put forward a line of thought that is neither negatively destituent (post-Heideggerian), nor affirmatively constituent (post-Deleuzian, post-Spinozian), but instituent (neo-Machiavellian), capable of thinking about order through conflict (the affirmative through the negative). Instituting should therefore be conceived as producing innovation *not* starting from nothing (according to the political theology of *creatio ex nihilo*), but starting from an *already* established reality, where the past is the negative that must be integrated and radically renewed through ever new institutions.

This broad theoretical premise allows me to more clearly address the problems from which I started, while also taking into account their political implications.

### Europe and philosophy

I will therefore start with the first question, namely the link between Europe, philosophy and politics: Esposito, in all his contributions regarding the European question, emphasizes the deep bond that exists between Europe and philosophy, and, not surprisingly, speaks of “Europe’s philosophical vocation.”^18^ In his view, it is precisely this philosophical vocation that should be the starting point for devising a new articulation of its structure, as has happened in the past, especially in times of crisis: “Why not imagine even in the current situation–in which Europe is threatened in its very form of life–that philosophy can indicate, if not a solution, at least a new way of seeing things and a direction to take?”^19^ Today, for example, in the absence of political unity, Europe is in a stalemate: “This stalemate is precisely what opens up a new space for philosophical reflection–not because the latter has solutions at hand for highly complex problems but because, in times of drastic changes on the world scene, philosophy may be in a better position than other types of discourse to recognize beforehand the direction the events are taking.”^20^ The crisis can obviously also be interpreted by other human sciences, “but when every point of reference is in a process of change such as the one we experience today, only philosophy is capable of grasping them together as a whole.”^21^ The intrinsic link between Europe and philosophy is therefore the horizon within which we must remain.

Philosophy should also be viewed in the context of its dialectic tension with politics. Indeed, Esposito also considers the dialectic between philosophy and politics to be integral not only to the idea of Europe, but also to its very existence in history: Europe is perceived both as a *part* of the world and as a very precise *perspective* from which to look at it. The wars that have afflicted Europe, sparked by a desire to extend its borders, have contributed to the development of ever-deeper self-awareness. Hegel previously argued this point in relation to the wars between the Greek cities and the Persian Empire. It is in this historical contingency that Europe, and therefore the West, has become aware that it is not simply one of the cardinal points, but a geo-political space: “Since its inception, European life has been inextricably intertwined with political affairs and the work of thought. While the latter has had a tendency to objectify itself in institutional forms from the outset, politics, by contrast, separates itself from naked violence, taking shape based on rational assumptions.”^22^ There consequently exists a point of tangency between Europe, philosophy and politics, between *logos* and *polis*, between thought and institutions. Not surprisingly, the political crises that Europe has endured have always been accompanied by reflection that appears to become more fruitful and productive in times of crisis. However, according to Esposito, it was profoundly misleading to attribute Europe’s political crises, especially from the nineteenth century onwards, to a crisis of its tradition of thought, as if the political crisis stemmed directly from a philosophical crisis.^23^

Esposito’s thesis is that it was, above all, lack of awareness about the importance of what lies “outside” philosophy that led European philosophy in the first half of the twentieth century to an impasse. Philosophers should have focused not only on the philosophical horizon, but on its point of tangency with what lies *outside* it, namely, in particular, on the *historical and political situation*. Reflecting on the proposals of Husserl, Heidegger, Ortega and Valéry, Esposito notes that the belief in consubstantiality between Europe and philosophy–which unites these proposals–results in the crisis of one being identified with that of the other: a metaphysical crisis whose remedy is a return to Greek origin. According to Esposito, other interpretations of the crisis though based on a comparison with the “outside” fail to give a satisfactory philosophical-political answer. Despite the originality and fecundity of their positions, other authors who fail to convincingly think about a fruitful relationship between philosophy and politics are, for example, on the German front, Adorno and Horkheimer, who arrive at a tragic negativity with no solution in sight, and on the French front, Derrida, who remained a prisoner within a decidedly *unpolitical* horizon.^24^ In contrast to the perspectives developed in Germany and France, Italian thought has always tried to tie philosophical and political thought together.^25^ This distinctive characteristic of Italian thought has meant that categories that certain Italian thinkers have worked on have become central to rethinking not only European politics, but also global politics: Negri’s *imperium*, Agamben’s *sacertas* and Esposito’s *immunitas*.^26^

Esposito’s thought still lies within this perspective, in which philosophy and politics are conceived together, as was clear already from his research on the *unpolitical*.^27^ But also on the *origin* of politics, which has been at the center of his reflection since the Nineties, during which he has focused on the thought of Hannah Arendt and Simone Weil.^28^ The same thing can be stated about his books on *community*, *immunity* and *biopolitics*.^29^ His thoughts on Europe and on the relationship between politics and negation stem from the belief that the lack of answers to the European crisis is closely linked to another matter concerning the *language* and *political categories* involved.^30^ In particular that of *sovereignty*, which has unanimously been considered central to reflections about the European crisis. One could argue that the notion of sovereignty is not a central one for *all* contemporary European thinkers. Indeed, just to mention an example, the Marxian tradition has developed a line of thought, which cannot be reduced to theological-political categories like sovereignty. I believe, in this respect, that Esposito’s critique of the notion of sovereignty owns a lot to his confrontation with other Italian thinkers belonging to the large area of post-marxian thinkers, which is itself quite rich and differentiated.^31^ I am thinking, in particular, to the dialogue between Toni Negri and Esposito on the role of Italian philosophy.^32^ Also, the lively and important debate between Negri and Esposito in 2016 revolves exactly around the issue of *sovereignty*.^33^ Their points of view are certainly quite distant, but I would not define them as “in contrast,” rather “in divergent agreement,” as one could see from Esposito’s words on the intellectual history of Toni Negri.^34^ But also, in my opinion, from Negri’s answers to Esposito’s questions.^35^

According to Esposito, the limitation of the category of *sovereignty* is that it is completely inadequate for representing the *biopolitical* dynamics that have impacted the whole of Europe and the rest of the world. We need only to consider two of the problems that Europe must currently contend with: the migrant problem and the widening gap–both in individual countries and in Europe as a whole–between those who Esposito calls “the two peoples of Europe,”^36^ one formed by the most affluent and the other composed of the growing and wide-ranging mass of poor people. With regard to the first problem, Esposito believes that the migrant humanitarian crisis calls into question the profound meaning of Europe’s existence: “Without exaggerating the importance of the ultimate question regarding their fate, they can be kept alive or left to die. The meaning of what we call Europe also depends to some extent on how it responds to this radical alternative.”^37^ It is therefore a crisis of destiny “on the precarious borderline that separates an affirmative biopolitics from a thanatopolitical crisis of unknown proportions,”^38^ according to the terms still used by Esposito in 2016, which, as we shall see, seem to remain in the background in the latest reflections.

In reference to the second issue, namely the growing gap between rich and poor in Europe, Esposito observes how only one of the two peoples is already represented within European institutions, namely the richest, through lobbies and global finance. The most urgent need is therefore to give representation to millions of poor people who currently do not have a voice in European institutions. Thus, “the process of Europe’s political unification will not be the fruit of agreements between summits, but the result of a real political dialectic.”^39^

## Rethinking negation and political ontology

This “misdiagnosis”–the attribution of Europe’s political and institutional crisis purely to a crisis of thought–leads us to the second point, namely the analysis, however necessarily synthetic, of the inadequacy of the response of certain ontological-political paradigms and certain theological-political categories to the European crisis and, within them, to the fundamental role played by the category of *negation*.

So why does philosophy–as well as the Left–find itself in a stalemate in terms of its answers to the current crisis? In his 2019 work *Politics and Negation* Esposito turned his attention to the question of *negation*, which is given even greater consideration in his most recent contributions.^40^

In this regard, I think it is necessary to add a side note on Esposito’s approach. Not infrequently, concepts that emerge as “cornerstones” of his thought at a given time have already played an important role. With regard to negation, for example, the productive role that it played in *Immunitas* was evident through the concept of “immunitary tolerance.”^41^ Negation played a central role, as I have previously mentioned, also in his theological-political thought, whose declared objective was to depart from the semantics of negation towards an affirmative ontology that would lead beyond theological-political categories.^42^ It seems to me, therefore, that one of the characteristics of his philosophical approach is its tendency to evolve in a “spiral-like” manner, shining a spotlight, prompted by the urgencies of the present, on what constituted the background of some of his philosophical proposals in the past. If we do not understand this approach, which entails successive focuses and, at the same time, retroversions, we might be surprised by what, at first glance, necessarily appears to be a radical change of both philosophical and political perspective. Where, for example, should we look for the turning point that led the philosopher of “affirmative biopolitics” to the horizon of “instituent thought” (neither destituent, nor constituent)? The philosophical answer can only be traced to the different focus on the question of negation, through a different reading of its relevant authors. Indeed, Esposito’s thought frequently emerges between the lines of his hermeneutic work on other authors and it is therefore in the dialogue woven with these authors that I believe it will be possible to best follow the development of his ideas. Also in relation to negation (and affirmativeness) we can trace the change in this way: philosophy’s change of direction from a tragic negation–like that which Adorno arrives at, which gives the question of the negative a central role, so much so as to make it the pivot around which his entire philosophical-political thought revolves–to a *purely affirmative* philosophy, like that of Deleuze’s last works, had also produced a break with the Hegelian dialectic between affirmation and negation, to attest to a *pure* affirmation.^43^ The *plane of immanence* is, for Deleuze and for the philosophers who share his perspective, the exclusive horizon in which power and potentiality are inextricably linked by a double thread.

Esposito traces an oblique path between these two *excesses*: we cannot remain crushed within the theological-political machine of negation, but neither can we claim a radically affirmative ontology and politics, in which every reference to the negative disappears. Essentially the question to be answered is: can the two poles of affirmation and negation be maintained without making their relationship assume a dialectic trend that would necessarily lead back to the prevalence of one or the other? In order to pursue this path, Esposito, first of all, broadens the horizon of reflection on negation: “rarely do studies on the function of the ‘not’ in linguistic denotation connect with work that has been done in logic on the judgment of attribution, or with ontological theory on the status of nothingness.”^44^ His research precisely sets out this articulation, in that, in his opinion, it is only this way that we can understand the consequences of the still unexpected semantic shift of the negation from the logical level to the ontological level and, finally, to the political level: “the transition of the negative from a linguistic to a logical use, and from this to an ontological one, to arrive at a performative use is exactly the same passage through which its metapolitical effect can be understood.”^45^ From a political point of view, on the other hand, there has been no reflection on “the structurally negative character of modern political categories,”^46^ a study that Esposito conducts genealogically, convinced that the politicization of negation has led to the negative trend in modern politics, still fully inscribed in the theological-political apparatus, with the prevalence, in its categories, of the negative over the positive, culminating in Nazi thanatopolitics.

To open a gash in political theology, one must not be crushed by the *negative* (as is the case, for example, in Adorno), but neither is it enough to simply “deny the negative,” assuming an entirely *affirmative* lexicon (as in Deleuze’s last works). Instead, it is necessary to *intersect* the negative, reinterpreting it within categories that can offer it a philosophically and politically more productive articulation: *difference*, *determination* and *opposition*.^47^ These are the key notions with which *Politics and negation* ends:

The value that Machiavelli gave to conflict as a central mechanism operating inside order; Kant’s concept of the real opposition between contrary forces, both of which are positive; the affirmative dialectics between action and reaction as Nietzsche conceived of it; the co-belonging of power and resistance theorized by Foucault; the understanding of immune processes as an internal threshold of community–all these are ways of understanding and practicing the “affirmativity” of the negative.^48^

*Difference*, *determination* and *opposition* represent alternative theoretical paths to philosophical thinking about politics, compared to those, inherited from political theology, of *enmity*, *exclusion* and *annihilation*. Through these “positive” notions of negation, Esposito envisages a possible escape from the paralysis of politics which, in his opinion, is determined both by tragic and untranscendable negativity (Adorno) and by affirmativeness without residue (Deleuze).

In Esposito’s latest articles–which are probably a preview of the book on political ontology that he is currently working on–his position with respect to negation is clear: “If absolute negation does not even allow us to imagine a praxis of transforming reality, an equally undifferentiated affirmation renders it useless because it is already implicit in the becoming of being.”^49^ Beyond the Adornian tragic, the Heideggerian unpolitical, the Deleuzian absolute affirmative: this is the horizon of Esposito’s latest research.

The perspective of the Foucauldian “ontology of the present,” which Esposito has considered for some time to be the perspective most suited to his approach to philosophy, led him, in his most recent research, to reflect on “political ontology.” Ontology, in this binomial, appears to be inextricably linked to politics and suggests a structural link which, according to Esposito, exists between *being* and *politics*. The question “what is politics (or the Political)”? spans the reflections of the greatest philosophers of the twentieth century, from Schmitt to Lefort, although the implication between being and politics was already evident in Plato’s writings. Esposito does not, of course, address a *foundational* ontology, but rather speaks of “an ontology of difference and not of identity, of the outside and not of the inside; of an ontology cut inside by an original partition, of Two rather than of One.”^50^ Esposito views the link between ontology and politics as an obvious fact, given that philosophy *always* involves a certain vision of the world which, naturally, *also* translates into a certain political vision. On the other hand, events that we might describe as strictly political lead, not infrequently, to rethinking conceptions of a certain being of the world, as happened with the religious wars, the French Revolution and the protests of 1968: political upheavals that aroused deep questioning even in the recognizable movements of thought that lay behind them. Therefore, *a* sense of history, a single meaning in which to encompass different phenomena, is not at stake. The question is, rather, that of assuming an obvious central fact: in summary, on the one hand, philosophy *always* involves a certain vision of the world which, naturally, translates into a *certain* political vision. On the other hand, certain political events lead to rethinking a *certain* being of the world. It is therefore necessary for thought to grasp the *emergence* of a certain *meaning*, of a certain *sense* that can become dominant in a given historical period and marginal in another: “in this sense history, great history, has become the living material of philosophy, while philosophy itself has become a constitutive form of contemporary history.”^51^

The philosophers who, in the twentieth century, explored the status of the Political with a particularly radical approach, from Schmitt, to Arendt, to Foucault, all asked themselves a question about the being of politics and power, without placing themselves in a metaphysical horizon. Indeed, this question arises from awareness “of the necessarily ontological character of thought on politics.”^52^ According to Esposito, what differentiates the various ontological-political paradigms is the relationship which, within them, is produced between being, difference and politics: the multiple combination of these three terms can in fact change their meaning and the horizon within which a given political ontology can be placed: “Politics can trace within itself a bar that reproduces the ontological difference on a different plane. Or constitute the inherently differential character of a being lying on a single plane of immanence. Finally, according to another register, being, in its social dimension, can be established by a symbolic difference that has the divisive characteristics of politics.”^53^ This triangulation respectively connotes the ontological-political paradigms which Esposito discusses, namely the Heideggerian and Deleuzian paradigms, and characterizes the *neo-Machiavellian* one proposed by Esposito.^54^ Very different lines of thought, such as those of Arendt and Marcuse, for example, can be ascribed to the *post-Heideggerian* paradigm, despite their eventual detachment from the reflections of Heidegger which provided their starting point, as well as those of Nancy and Agamben. In this case, the profound difference in their political visions should be attributed, according to Esposito, to the different relationship with the “unpolitical” background of Heidegger’s thought, already evident from his conception of the *polis*, which became predominant in Heidegger’s last works, with a clear prevalence of the *negative*: politics, in fact, was identified with the destructiveness of the technique.^55^ The *unpolitical* outcome of this paradigm emerges, for example, in the concept of *deactivation*, at the origin of a *destituent* tendency, proposed, in particular, by Agamben, with his theory of *destituent power*, as well as by Nancy, with the category of *inoperativeness*.^56^

The second ontological-political paradigm is the *Deleuzian* paradigm, which is rooted in Spinoza, Nietzsche and Bergson. In it, in contrast to the Heideggerian paradigm, a clear prevalence of *affirmation* and the evident centrality of a *plane of immanence* emerges. Despite Deleuze’s strong criticism of Heidegger, Esposito underlines the fact that they have one point in common, namely the ontological aspect attributed to the political through an approach that focuses on *difference*.^57^ However, what Esposito calls “the triangulation between politics, being and difference”^58^ is also what most divides them: indeed, Deleuze excludes the difference between being and beings and therefore everything that does not lie on a plane of immanence. For Deleuze, *Being* itself is *Difference* and therefore has a *constituent* power, opposed to any *destituent* power: it continually creates, indeed it creates *itself*, as difference. There are no assumptions, but only the *movement* of *generating* in its pure affirmativeness, as is evident, for example, in Antonio Negri’s paradigm of constituent power.^59^

This does not mean that negation *disappears* for Deleuze: “Even when he disputes the negative, he does not deny its presence, but, by translating it into difference, he repositions it in a positive perspective. Only a completely superficial interpretation of Deleuze can ignore the dramatic, and even tragic, tone that disturbs his entire work like a bottomless pit in which it is possible to slip.”^60^ In fact, Esposito’s critique concentrates above all on Deleuze’s last works, in the wake of Badiou: focusing political philosophy on analysis of capitalism, making it the most powerful desire production machine, leads Deleuze *not* to oppose it, but to *accelerate* its dynamics, pushing it to implode, in an analysis, however, in which an all-pervasive politicization seems to dominate; yet if *everything* is political, *nothing* is political.^61^ So how can we distinguish the vanishing lines of emancipatory politics from those implemented by capital? Do we not risk indifferentiation in which it is impossible to access the conflict line?

This *indifferentiation* is the threshold on which Esposito distances himself from Deleuze’s last works, from which, however, he recovers certain elements that are useful for developing his own neo-Machiavellian paradigm. Deleuze’s last works are no longer a source of inspiration, but rather his first works, in particular his somewhat unique text *Instincts and Institutions*.^62^ A more “political” Deleuze, in which negation still played a dialectic role in relation to pure affirmativeness.^63^

## Towards instituent thought

The political ontology on which Esposito focuses in his latest research is, in fact, that in which affirmation and negation are *both* maintained, without, however, slipping into the dominance of one over the other or, worse, into the neutralization of one of the two terms.

The favored “political space” for this productive co-implication of affirmation and negation is the *institution*. It is first and foremost in the Deleuzian interpretation of certain classic texts on the institution that Esposito identifies the possibility of a “dynamic” rereading of the institution itself, referring to other scholars–from Castoriadis to Lefort, Dardot and Laval, just to name a few–who view the institution from a *non-static* perspective.^64^ How? Through what we could define as its “desubstantivization” and its increasingly verbal nature. A passage from noun to verb: rather than “institution,” “to institute,” rather than a fixed product, a continuous praxis, giving life to the new not as *creatio ex nihilo* (in the manner of a theological-political sovereign decision), but as an innovative “recovery” (to borrow a term from Merleau-Ponty) of what exists. Such a shift from noun to verb emphasizes the *becoming* of the institution and its ability to change its characters. This concept of institution represents a deviation from what is perhaps its closest paradigm, namely “constituent power.” This is not a case of *creatio ex nihilo* as in the constituent paradigm. In order to think and act in an *instituent* manner, a space is needed in which the different institutions are already present. In their *substantival* form, they constitute the negative that comes into tension with the predicate of instituting. Order and conflict are not, in this horizon, alternative, but are held together dynamically. Reflecting on the role of *conflict* has been a recurrent theme in Esposito’s thought. Indeed, already in the Nineties, while thinking about the *origin* of politics, Esposito had opened an intense and fruitful confrontation with Hannah Arendt and Simone Weil, two thinkers that in his more recent work appear to have a marginal role. As Esposito correctly pointed out, both Weil and Arendt identify the Trojan War as the origin of Western history and Western politics; a war that ends with the total destruction of Troy: “Politics in this sense, is born at the heart of a *polemos* whose outcome is the destruction of a *polis*. It is upon this constitutive antinomy that the two authors measure themselves, fully aware of what it means not only in relation to the reconstruction of the initial event itself, but also in relation to the interpretation of everything that follows.”^65^ Central to Arendt and Weil’s vision, at least in Esposito’s own interpretation, was not the theme of what *institutions* could possibly confront such a disquieting origin, but rather the ontological question on the origin of politics itself: “It is this bond between origin and politics–the political destiny of the origin but also the constitutive originarity of politics–that captures the attention of both thinkers, who had already made the *polis* the primary concern of their reflection. […] The question to be resolved is, precisely, that of relationship between origin–a specific originarity–and what originates from it.”^66^ Esposito’s most recent perspective, on the other hand, poses an indissoluble bond between ontology and politics, but with a very special attention to the specifically political consequences of every ontology.

It is therefore no surprise to find in this context the other author who Esposito has never ceased to discuss, from the outset, establishing a dense, productive dialogue, namely Machiavelli, who does not believe that the institution imposes a clear alternative between order and conflict. It is no coincidence that Esposito characterizes the ontological-political paradigm on which he is working with the adjective “neo-Machiavellian.” Esposito therefore chooses to dialogue with certain contemporary authors, whose interest in Machiavelli is evident. In particular Claude Lefort because, in his opinion, Lefort best expresses the role of difference–in the triangulation between being, politics and difference–in an instituent sense.^67^*Difference* for Lefort “is the break that politics imprints on a social being that is still unaware of its original division. […] In the perspective that can be defined neo-Machiavellian, the difference is the political institutionalization of a society that has always been separated from itself, albeit in an unreflective way.”^68^ The theoretical nucleus that convinces Esposito of the current importance of Lefort’s proposal is the fact that, for him, the social must be established not according to a *conservative* paradigm of the institution, but re-evaluating the role of conflict, which has always been part of the social domain, through its expression on a *symbolic*, rather than *factual*, level. The institution, therefore, is not exclusively viewed as a *limitation* of conflict but, rather, it must be expressed in conflictual terms: “Generated by original social conflict, the instituent act has the task of reproducing it, legitimized and strengthened by the political decision.”^69^ Machiavelli is the source of inspiration of this particular conception of the instituent act, given that in his thought the principle of struggle is not comparable to that of antagonism, as Merleau-Ponty had already noted.^70^ Rather, conflict is a *condition* of the community and, in this sense, Esposito believes that a convincing and productive political ontology emerges from the Machiavellian interpretation of Lefort: in the relationship between the Prince and power Lefort sees a representation of man’s relationship with Being. The social, from this perspective, can never *directly* relate to itself, but “can only be recognized by pushing the gaze towards something–namely power–that is installed outside it.”^71^ There is therefore a return of the constitutive relationship with the negative, with division, which is the condition of social unity, “in the double sense of the distance of the power that establishes it and of the conflict that cuts it from within.”^72^ Far from leading to a *dismissal* of the already established, the conflict thus imagined relates to it in a *non*-predeterminable dynamic, acting on a social unit that pre-exists and can be, from time to time, changed with the aim of constantly improving the institutions themselves.^73^ Therefore, Claude Lefort maintains that the main virtue of institutions is to ensure that conflict does not turn into pure *antagonism*, but remains on a *symbolic* level: democracy is founded on this principle. Indeed democracy, Esposito stresses, in reference to Lefort, is “kept alive by an institutionalized conflict that makes the place of power empty, that is to say always contestable […]. It does not belong to anyone, not even to the people.”^74^

This is the challenge of instituent thought: to try to think of a conflict that produces order and social unification starting from an ineliminable division. But it is precisely this–a challenge–the outcomes of which are not predictable, since they are always at risk of descending into absolute antagonism and into usurpation of the place of power by forces external to politics, such as the economy. Nonetheless, it is with this old, yet always new challenge that Esposito attempts to contend.

## Footnotes

1 Esposito (2019c).

2 Esposito (2018a).

3 Esposito (2015a).

4 Regarding the broad debate on political theology, see Agamben (2011), Benjamin (1986), Blumenberg (1985), Cacciari (2018), Fulco (2007, 2017), Kantorowicz (1997), Lefort (2006), Moltmann (1999), Peterson (2011), Reinhard (2005), Rorty and Vattimo (2005), Schmitt (2005), Stimilli (2017b), Stimilli (2019), Strauss (1967), Taubes (2003).

5 Esposito (2015a, pp. 1–2).

6 On this see, in particular, Schmitt (2005), Peterson (2011), Taubes (2013).

7 Esposito (2015a, p. 3).

8 In particular, see Schmitt (2007).

9 Esposito (2019c, p. 19). For a deconstruction of this Schmittian paradigm, see Derrida (1997), but also Resta (2008).

10 About the concept of *person*, see Esposito (2012a, 2018b).

11 As he states at the end of the first chapter, Esposito wants to try to offer a resistance to the theological-political lexicon through the deconstruction of the apparatus on which it is based, namely that of *person*, in order to conceive of a way to overcome the *katechon* without absolute negation of the *apocalypse*: “What is involved is nothing less than imagining a Parousia without Apocalypse, or an affirmation without negation” (Esposito 2015a, p. 82).

12 Esposito (2015a, p. 192).

13 Esposito (2015a, p. 195).

14 Esposito (2007). See, on this topic, also Campbell (2007), Forti (2017), Esposito (2017a), Resta (2017).

15 Esposito (2013), (2018c), (2019b). Certain themes that are key to reflection on political ontology are, however, already present, for example in the two essays *Che cos’è la filosofia* and *Politica e metafisica*, both in Esposito (2018c).

16 Esposito (2020, 2021).

17 On the persistence of borders and on the ambivalence of globalization, see Resta (2020, pp. 206–219).

18 One may hold the view that Esposito’s description of the link between Europe and philosophy as “essential” is itself the outcome of a form of circular reasoning: an idea too much influenced by a euro-centric view of the history of philosophical thought. This view could reasonably be held by those that interpret the word “philosophy” as synonym for “cultural tradition.” If this premise is accepted, indeed one may argue that even other geo-political entities maintain an “essential relationship” with cultural and religious traditions which shaped their history: think of India, China, Japan. Still, one should keep in mind that in this context Esposito—like Hegel, Heidegger, even Derrida, have done before him—is not talking about generic “cultural traditions,” but about that very specific form of thought, first developed in Greece, which indeed is indistinguishable from the history of Europe, as firmly stated, once and for all, by Heidegger in a memorable conference in Cerisy-la-Salle, in 1955: “The word *philosophia* tells us that philosophy is something which, first of all, determines the existence of the Greek world. Not only that–*philosophia* also determines the innermost basic feature of our Western–European history. The often-heard expression ‘Western-European philosophy’ is, in truth, a tautology. Why? Because philosophy is Greek, in its nature; Greek, in this instance, means that in origin the nature of philosophy is of such a kind that it first appropriated the Greek world, and only it, in order to unfold” (Heidegger 2003, pp. 29, 31). The choice of considering philosophy as an essentially western development, did not prevent many intellectuals sharing such a view from fighting Europe’s political and cultural colonialism. In this respect Franz Fanon’s work is, in my opinion, emblematic, and, one should not forget, it had the support of an intellectual and philosopher like Sartre: see Fanon (2004).

19 Esposito (2018c, p. 86).

20 Esposito (2018a, p. 2).

21 Ibid*.*, p. 2.

22 Esposito (2018c, p. 85).

23 On this question, see also Di Cesare (2018).

24 The political vocation of deconstruction has been highlighted by Bennington (1994), Resta (2003, 2016, 2017).

25 Esposito already does so starting with his first books in which he discusses, for example, Machiavelli and Vico: see Esposito (1980, 1984).

26 See Calcagno (2015), Gentili and Stimilli (2015), Lisciani and Strummiello (2017), Stimilli (2017a). A relevant, quite recent, use of the concept of *immunitas* is the one brought about by the exceptional situation caused by the Covid-19 pandemics, see Esposito’s interview with Christiaens-De Cauwer (2020). This renewed interest is to be contrasted with Han’s critical appraisal of the immunitarian paradigm (see: Han 2015). On this issue, see Wyllie (2020).

27 Esposito (2015b). On this concept see also Gentili (2012).

28 Esposito (2017b).

29 Esposito (2010, 2011, 2007). On this very important part of Roberto Esposito’s thought, see Viriasova and Calcagno (2018), Bird (2016).

30 In addition to Esposito (2018a) see also the second part of Esposito (2018c), entitled *Pensiero italiano e filosofia europea*.

31 An important moment of discussion starting from Esposito’s position on the Italian thought took place in Belgium in 2018. A lot of attention was devoted also to views critical of Esposito’s own approach and more generally of the different components of the Italian thought (e.g. Toni Negri, Franco Berardi, Giorgio Agamben, Paolo Virno, Massimo Cacciari, Mario Tronti). See Tim Christiaens and Guilel Treiber (eds.) *Italian Philosophy and the problem of potentiality* (https://hiw.kuleuven.be/cespp/events/agenda/workshop-italianphilosophy-potentiality). On the issue of the reception and discussion of Esposito’s theses, and more generally of the Italian thought, in Belgium, see Christiaens (2019).

32 See Negri (2016), Esposito (2012b, in particular, pp. 231-233).

33 This lively debate took place during the first DeriveApprodi festival. It was clear to each of us present at the meeting that we were witnessing an “epoch-making” confrontation about issues that are central for our appraisal of the present times (see: https://www.deriveapprodi.com/2016/10/il-primo-festival-di-deriveapprodi/).

34 Esposito (2017c). See also what Esposito says about the notion of “constituent power” in Toni Negri’s thought: “Come per Machiavelli, il potere costituito non si emancipa mai del tutto da quello costituente, ma ad esso ritorna per rinnovarsi radicalmente ogni volta che rischia di prosciugarsi. Da questo punto di vista, in ordine a questa opzione di fondo anti teologico-politica, sono d’accordo con lui e da tempo mi muovo anche io nella stessa direzione. Ciò che mi convince meno è la sua interpretazione della situazione attuale” (ibid. p. 25). See also, ibid., p. 24 and p. 31.

35 Negri (2017, pp. 33–40). Both Esposito’s and Negri’s contributions are so rich and stimulating that, to be able to discuss them correctly, one would need to write a separate article.

36 Esposito (2018a, pp. 221–232).

37 Ibid., p. 3. About the relationship between Europe, institutions and migration see Di Cesare (2020), Fulco (2019a).

38 Esposito (2018a, p. 3).

39 Ibid., p. 232.

40 Concerning the development of the notion of negation, it seems to me that even in this case, like in the one of sovereignty, a fundamental role was played by Esposito’s confrontation with other post-marxian thinkers. One could for example mention the important book by Paolo Virno (2018). A book that, in Esposito’s own words, was a decisive inspiration for his work on this notion: see Esposito (2019c, 2. *Negative terms,* par. 5).

41 Esposito (2013, p. 134).

42 The question regarding a positive form of the political returns urgently in Esposito (2019c): “But is this negative and exclusionary relation between metaphysics and politics the only one possible; or is it possible to imagine another relation that responds to positive and inclusionary needs? In other words, is an affirmative metapolitics conceivable?” (ibid., p. 128).

43 On Adorno see, in particular, Esposito (2018a, pp. 66–72).

44 Esposito (2019c, p. 1).

45 Ibid., pp. 1–2.

46 Ibid., p. 2.

47 Not surprisingly, Esposito dedicates the second part of the volume to these notions, see Esposito (2019c, pp. 137–199).

48 Esposito (2019c, pp. 8–9).

49 Esposito (2019a, p. 10).

50 Ibid., p. 8. The main reference for a post-foundational ontology is Marchart (2007).

51 Esposito (2019a, p. 8).

52 Ibid., p. 8.

53 Esposito (2019b, p. 25).

54 Esposito focuses on the Heideggerian paradigm in Esposito (2019b, pp. 25–29). On the Deleuzian paradigm, see Esposito (2019b, pp. 29–34). On the Machiavellian one, see Esposito (2019b, pp. 34–39). It is important to emphasize that Esposito does not view these paradigms as a chronological sequence, but rather in terms of their *co-presence* in the fabric of history and politics.

55 Esposito refers, in particular, to the Heideggerian reflections contained in Heidegger (1992): see Esposito (2019b, p. 27). On this question, see e.g. Fistetti (1999).

56 See e. g. Agamben (2016), Nancy (1991).

57 In particular, see Deleuze (1994).

58 Esposito (2019b, p. 31).

59 See Negri (1999).

60 Esposito (2019b, p. 32).

61 See Badiou (2009).

62 Deleuze (2004). On Deluze’s concept of institutions, see, among others: Fadini (2019).

63 On this see also Bazzicalupo (2019, pp. 90–92).

64 Deleuze’s thought is certainly a source of inspiration, but other figures have played an important role in defining the nature of Esposito’s instituent thought, including Castoriadis (1997), Douglas (1986) or, as we shall see, in particular Lefort (2006, 2007), Lefort and Gauchet (1971, pp. 7–78). But also, jurists like Romano (2019) or Hauriou (1970). On the question of *institutions*, in an interesting discussion with Esposito’s thought, see: Lisciani and Adinolfi (2019).

65 Esposito (2017b, p. 13).

66 Ibid. For a more detailed analysis of the questions addressed by Esposito in his confrontation with Arendt and Weil, see Fulco (2019b). In his more recent work, Esposito classifies Weil in the “destituent paradigm,” see Esposito (2020, pp. 23–24; pp. 51–52). For an interpretation of conflict and vulnerability in Simone Weil, taking into account also Esposito’s more recent reading, one could refer to Fulco (2020b, especially p. 43 and pp. 130–131).

67 The volume that Lefort dedicated to Machiavelli is also important in this reflection: Lefort (1972).

68 Esposito (2019b, p. 35).

69 Ibid., p. 35.

70 See e.g. Merleau-Ponty (1960).

71 Esposito (2019b, p. 37).

72 Ibid., p. 37.

73 Levinas’ thought represents a position that is simultaneously close and divergent to this. Levinas, while envisaging a path towards “institutions that are always just,” believes that it can be accomplished not so much starting from *conflict*–which, in “left to itself” politics will always declare the victory of the “strongest”–but from the *tension* between an *inside* and an *outside* the institutions, between *politics* and *ethics*, instances that, within truly democratic institutions, should be considered *heterogeneous* but, at the same time, *inseparable*. On this question, see Fulco (2013, 2020a). Esposito gives Levinas this *ancipital* status that places him in an ontological-political paradigm that cannot be traced back to the prevalence of pure negation, nor to that of affirmation without residue: “Moreover, the terrain of the philosophical discussion of the second half of the 20th century cannot be reduced to the dry alternative between negative thought and affirmative thought. Authors such as Derrida and Levinas, for example, escape this dichotomy, placing themselves in the line of tension between the two opposing fronts” (Esposito 2019a, p. 9).

74 Esposito (2019b, p. 38).
